# Genesis of a novel *Shigella flexneri *serotype by sequential infection of serotype-converting bacteriophages SfX and SfI

**DOI:** 10.1186/1471-2180-11-269

**Published:** 2011-12-30

**Authors:** Qiangzheng Sun, Ruiting Lan, Yiting Wang, Jianping Wang, Xia Luo, Shaomin Zhang, Peijing Li, Yan Wang, Changyun Ye, Huaiqi Jing, Jianguo Xu

**Affiliations:** 1 State Key Laboratory for Infectious Disease Prevention and Control, National Institute for Communicable Disease Control and Prevention, China CDC, P.O. Box 5, Changping, Beijing, China; 2 School of Biotechnology and Biomolecular Sciences, University of New South Wales, Sydney, NSW 2052, Australia

## Abstract

**Background:**

*Shigella flexneri *is the major pathogen causing bacillary dysentery. Fifteen serotypes have been recognized up to now. The genesis of new *S. flexneri *serotypes is commonly mediated by serotype-converting bacteriophages. Untypeable or novel serotypes from natural infections had been reported worldwide but have not been generated in laboratory.

**Results:**

A new *S. flexneri *serotype-serotype 1 d was generated when a *S. flexneri *serotype Y strain (native LPS) was sequentially infected with 2 serotype-converting bacteriophages, SfX first and then SfI. The new serotype 1 d strain agglutinated with both serotype X-specific anti-7;8 grouping serum and serotype 1a-specific anti- I typing serum, and differed from subserotypes 1a, 1b and 1c. Twenty four *S. flexneri *clinical isolates of serotype X were all converted to serotype 1 d by infection with phage SfI. PCR and sequencing revealed that SfI and SfX were integrated in tandem into the *proA-yaiC *region of the host chromosome.

**Conclusions:**

These findings suggest a new *S. flexneri *serotype could be created in nature. Such a conversion may be constrained by susceptibility of a strain to infection by a given serotype-converting bacteriophage. This finding has significant implications in the emergence of new *S. flexneri *serotypes in nature.

## Background

*Shigella *is the primary pathogen causing bacillary dysentery in developing countries. There are an estimated 164.7 million people worldwide infected by *Shigella *annually; resulting in 1.1 million deaths, most being children under five years [1]. A more recent study estimated approximately 125 million annual shigellosis cases and 14,000 related deaths in Asia [2], suggesting that the death rate has decreased significantly in recent years. Among the four *Shigella *species, *S. dysenteriae*, *S. flexneri*, *S. boydii*, and *S. sonnei*, *S. flexneri *is the predominant species [3].

*S. flexneri *serotyping are based on structure of the O-antigen lipopolysaccharide. There are 15 known serotypes: 1a, 1b, 1c, 2a, 2b, 3a, 3b, 4a, 4b, 5a, 5b, 6, X, Xv and Y [4,5]. Except for serotype 6, all share a common tetrasaccharide backbone of repeating units of *N*-acetylglucosamine-rhamnose-rhamnose-rhamnose [6]. By adding glucosyl and/or *O*-acetyl groups to one or more of the sugars on the tetrasaccharide unit, various serotypes are formed. Serotype Y possesses the primary basic O-antigen without any modification of the tetrasaccharide backbone [6].

It is well known that *S. flexneri *serotype conversion is mediated by temperate bacteriophages [6,7]. Six different serotype-converting phages or prophages, SfI, SfII, Sf6, SfIV, SfV and SfX, have been identified and characterized [8-12], which can convert serotype Y to serotype 1a, 2a, 3b, 4a, 5a and X respectively [8-12]. Except for Sf6 which carries a single gene, *oac*, for acetylation of the O-antigen [13], the other phages carry three genes, *gtrA*, *gtrB*, and *gtr*_type _for O-antigen modification. The first two *gtr *genes are highly conserved and interchangeable in function, while the third *gtr *gene encodes a type-specific glucosyltransferase responsible for the addition of glucosyl molecules to sugar residue(s) on the basic O-antigen repeating unit [9,12,14]. These phages integrate into the *S. flexneri *host chromosome either at *tRNA-thrW *downstream of *proA *[15] or at *tRNA-argW *adjacent to *yfdC *[11]. Once integrated, the *int *and O-antigen modification genes are located at the opposition ends of the prophage genome, flanked by an *attL *sequence on the left and an *attR *sequence on the right [15].

Recently, untypeable or novel serotypes of *S. flexneri *from natural infections had been reported worldwide [5,16,17]. A novel serotype 1c was identified in Bangladesh in the late 1980s and was a predominant serotype in Vietnam and other Asian countries [16,17]. Serotype 1c was a result of modification of serotype 1a with addition of a glucosyl group by a cryptic prophage carrying a *gtr1C *gene cluster [18]. More recently, a new serotype named as Xv emerged in China, and replaced 2a to become the most prevalent *S. flexneri *serotype [5]. Although the antigenic determinant for the v variant is not yet known, the phage SfX, which is responsible for the group 7;8 antigenic determinant, was inducible from the sequenced *S. flexneri *Xv strain 2002017 [5]. Therefore emergence and spread of novel *S. flexneri *serotypes in nature poses a significant public health threat globally and in particular in developing countries where *S. flexneri *is the predominant cause of shigellosis.

In order to reveal possible roles played by the serotype-converting phages in the emergence of new serotypes, and potential of emergence of novel serotypes through this mechanism in nature, we performed infection assays using SfI and SfX, the 2 most common serotype-converting bacteriophages carried by *S. flexneri *based on serotype frequency data [5,19]. We demonstrate that a novel serotype, named serotype 1 d was created in laboratory by infecting *S. flexneri *serotype X strains with a SfI phage or by sequential infection of serotype Y strain with SfX and SfI.

## Results and discussion

### Creation of a new serotype, serotype 1 d, through serotype conversion with phages SfI and SfX

Using the procedures described by Mavris et al. [12], 2 serotype-converting phages, SfI and SfX, were induced and isolated from *S. flexneri *serotype 1a strain 019 and serotype Xv strain 2002017 respectively. The 2 phages were then used to sequentially infect a serotype Y strain 036 in different order.

We first performed sequential infection in the order of SfI and SfX. By infection with SfI, the *S. flexneri *serotype Y strain 036 was converted into serotype 1a (036_1a), which agglutinated with both diagnostic typing sera I and grouping sera 3;4 (also known as Y-5) as shown in Table 1. Strain 036_1a was then used for infection by SfX, but surprisingly, no plaques appeared, indicating the strain cannot be infected by SfX.

**Table 1:** Serological characterization of *S. flexneri *serotype Y, X, 1a, 1b and 1c using serotyping monoclonal antibodies (MASF)

Serotypes	Reaction with MASF										
	
	Type antigen specific						Group antigen specific				1c
	
	I	II	IV-2	V	VI	B	3;4*	6	7;8	IV-1	
Fy	-	-	-	-	-	+	+	-	-	-	-

Fx	-	-	-	-	-	+	-	-	+	-	-

F1a	+	-	-	-	-	+	+	-	-	-	-

F1b	+	-	-	-	-	+	-	+	-	-	-

F1c	-	-	-	-	-	+	-	-	-	-	+

F1d	+	-	-	-	-	+	-	-	+	-	-

*Y-5 is a synonym of grouping 3;4 antisera

**Figure 1:**
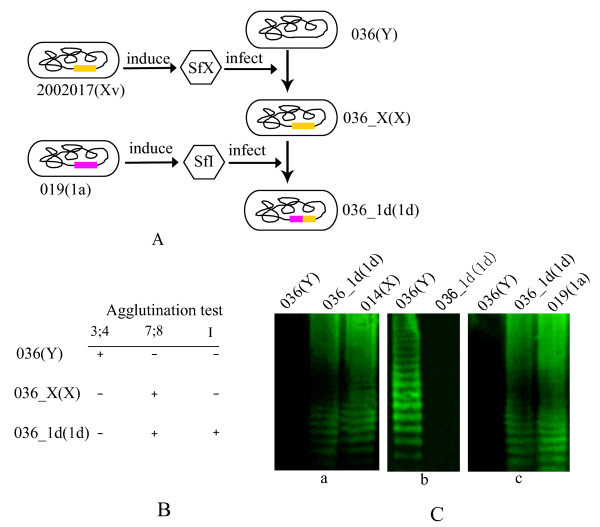
**Construction of a novel serotype, 1 d, of *S. flexneri *with serotype-converting bacteriophages SfX and SfI**. **(A**) Illustration of construction road map of *S. flexneri *036_1d strain from a serotype Y strain 036, by sequential infection of phages SfX and SfI. (**B**) Serological identification of *S. flexneri *036_1d as serotype 1 d with agglutination test using monovalent diagnostic sera. The constructed strain *S. flexneri *036_1d agglutinated with both of typing sera I and grouping sera7;8. (**C**) Serological identification of *S. flexneri *036_1d by Western-blot assay. The LPS extracted from the tested strains was separated by SDS-PAGE and hybridized with monovalent grouping sera 7;8 (**a**) and 3;4 (**b**), and typing sera I (**c**), respectively. LPS of serotype X strain 014 and serotype 1a strain 019 were used as positive controls for group specific antigen 7;8 and type specific antigen I. After strain name in brackets is the serotype of the strain.

Next we performed infection in the order of SfX and SfI. The *S. flexneri *serotype Y strain 036 was converted to serotype X by phage SfX infection, which agglutinated only with serotype X-specific grouping sera 7;8. We named this strain as *S. flexneri *036_X (Figure 1A and 1B). When 036_X was further infected with phage SfI, it was converted to a new serotype, which agglutinated with both of the diagnostic serotype 1a-specific typing sera I and serotype X-specific grouping sera 7;8, and were negative for all other type and group-specific sera (Figure 1A and 1B). The conventional serological identification results were further confirmed by Western-blot assay. As shown in Figure 1C, the lipopolysaccharide (LPS) pattern of the newly constructed strain was identical to that of serotype X strain 014 (Panel a, Figure 1C) and that of serotype 1a strain 019 (Panel c, Figure 1C), when probed by group specific sera 7;8 and type specific sera I respectively. Since the original serotype Y strain and its SfI convertant 1a strain can agglutinate with grouping sera 3;4, we also tested whether this antigen is detectable in serotype 1 d. The LPS of the new serotype was not recognized by the grouping sera 3;4 (Panel b, Figure 1C). Additionally, serotype-specific genes, *gtrX *for phage SfX and *gtrI *for phage SfI, were detected from these new strains by PCR and sequencing of the PCR products.

*S. flexneri *serotype 1 has three known subtypes, 1a, 1b and 1c, the agglutination patterns of which are defined by a combination of typing and grouping sera, namely typing sera I and grouping sera 3;4 (Y-5) for 1a, typing sera I and grouping sera 6 for 1b, *S. flexneri *group antigen specific MASF B and provisional specific monoclonal antibody MASF1c for 1c [17] (Table 1). Since the newly constructed serotype agglutinates with typing sera I, but showed a different serological pattern from all known serotype 1 subtypes (Table 1), we named this new serotype 1 d.

In order to determine whether such serotype-converting events could occur in nature, we randomly selected 24 *S. flexneri *serotype X strains in our collection, and infected them with serotype-converting phage SfI. All 24 strains tested were successfully converted to serotype 1 d.

We have no good explanation why serotype 1a strain 036_1a, constructed from 036 by infection with SfI, could not be further infected by SfX. We randomly selected 17 *S. flexneri *1a isolates from our collection for infection by SfX but found that none of them could be infected by SfX. Clearly, the SfI can infect the strains carrying serotype-converting phage SfX, but not vice versa, likely due to phage immunity from modified O-antigen receptors [20].

Interestingly, a recent study reported *S. flexneri *strains with identical serological characteristics to the novel serotype 1 d created in this study [21]. Four strains were designated as untypeable serotype I: (7;8) among 467 *S. flexneri *isolates collected in a passive surveillance project from Henan, China in 2006 [21]. Thus it seems that this novel serotype has already appeared in natural infections. Although serotype 1 d represented less than 1% of the isolates, it would be important to monitor this new serotype epidemiologically, considering that novel *S. flexneri *serotypes such as 1c and Xv achieved its dominance among the *S. flexneri *serotypes in a very short time frame [5,16,17]

### SfI and SfX integrated in tandem into the same site of host chromosome

**Figure 2:**
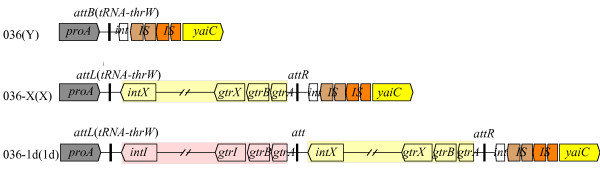
**Genetic organization of prophage genomes of SfX and/or SfI in *S. flexneri *036_X and 036_1d**. The prophage genomes of SfX and/or SfI are highlighted in yellow and pink respectively. The conserved genes of the host strain were shown in different colors: *proA*, gray; *yaiC*, yellow; IS600 ORF1 and ORF2, brown; IS629 ORF1 and ORF2, orange; the putative integrase gene (*int*), white. The integration sites *attB*, *attL *and *attR *are indicated in thick line. After strain name in brackets is the serotype of the strain.

It has been observed that the serotype-converting phages, except for Sf6, usually integrate into the *tRNA-thrW *gene of the host chromosome, which is adjacent to *proA *upstream [15]. However, the gene downstream the integrated phage have not been consistently identified [6,7]. Genomic analysis of *S. flexneri *serotype 2a strain 301 (NC_004337), 2457 T (NC_004741) and serotype Xv strain 2002017 (CP001383) showed that the serotype-converting phages were all integrated upstream of host gene *yaiC*. Thus cross-bridging PCR analyses of *S. flexneri *036, 036_X, and 036_1d across the *proA-yaiC *region were conducted using a series of primers and found that both phages SfX and SfI were integrated into the *tRNA-thrW *site, which is immediately downstream of gene *proA*, and upstream of gene *yaiC *(Figure 2). The phage SfI was found to be integrated immediately upstream of SfX genome, with an *att *site at both ends (Figure 2). By comparing the joining sequences between the serotype-converting phage genomes, we found that the phage SfI was integrated at the *attL *site of phage SfX (see Additional file 1). The integration site for the 24 serotype X isolates converted by SfI was also found to be the same site and thus it appears that the integration is very site specific.

## Conclusions

A novel serotype 1 d was constructed by sequentially infecting a serotype Y strain of *S. flexneri *with phage SfX and SfI, or by infecting clinical serotype X isolates with SfI. These results indicate that serotype conversion with phages SfI and SfX could occur in nature. However, the observation that the order of infection by the 2 phages affects convertibility of a strain indicates that serotype conversion is not only determined by the modification specific genes but also constrained by the properties of the serotype-converting phages. Our findings provide possible mechanisms how new serotypes of *S. flexneri *could emerge in nature.

## Methods

### Bacterial strains and phages

*S. flexneri *strains and serotype-converting bacteriophages used in this study were listed in Table 2. *S. flexneri *strain 036 (serotype Y) was used as host for phage infection and large propagation. *S. flexneri *strains 014 (serotype X) and 019 (serotype 1a) were used as positive controls in the serological assays for group specific antigen 7;8 and type specific antigen I respectively. Twenty four *S. flexneri *serotype X and 17 of *S. flexneri *serotype 1a strains isolated from patients and stored at National Institute for Communicable Disease Control and Prevention, China CDC (ICDC) were used for infection with serotype-converting phages SfI and SfX respectively.

**Table 2:** Strains and serotype-converting bacteriophages used in this study

Strains or phages	Relevant characteristic	Reference or source
*S. flexneri *strains		

036	Serotype Y	ICDC

014	Serotype X	ICDC

019	Serotype 1a	ICDC

036_1a	036 infected by SfI, serotype 1a	This study

036_X	036 infected by SfX, serotype X	This study

036_1d	036 infected by SfI and SfX, serotype 1d	This study

Phages		

SfI	Phage SfI, induced from *S. flexneri *strain 019	This study

SfX	Phage SfX, induced from *S. flexneri *strain 2002017	This study

ICDC, National Institute for Communicable Disease Control and Prevention, China CDC

Serotype-converting bacteriophages SfI and SfX were induced from *S. flexneri *serotype 1a strain 019 and serotype Xv strain 2002017 respectively, following the methods described by Mavris et al. [12].

### Phage infection and lysogen isolation

We used the procedures described for lambda phage (Φλ) for phage infection [22]. *S. flexneri *cells were inoculated into LB broth and incubated for 3 h at 37°C with aeration. Cells were harvested by centrifugation at 4000 rpm and the cell density was adjusted to 2.0 OD (A_595 nm_) with MgSO_4 _buffer (10 mmol/L). A proportion of cells (200 μl) were infected with purified phages with phage to bacterial cell ratio of about 1:1000 and incubated for 20 min at 37°C. The infected cells were mixed with 3 ml semisolid agar (Luria Broth (LB) with 0.7% agar) and immediately spread on LB solid agar plates. After incubation at 37°C for 20 h, the lysogens were detected from turbid single colonies.

### Slide agglutination and LPS analysis

Serological identification was performed using two commercial slide agglutination serotyping kits: monovalent anti-sera (Denka Seiken, Japan) and monoclonal antibody reagents (Reagensia AB, Sweden) according to manufacturer's instructions. The new serotype was further confirmed by Western-blot assay. Briefly, LPS was prepared using the method of Hitchcock & Brown [23] and transferred onto a PVDF membrane in a Tris/glycine/methanol buffer. The membrane was blocked with phosphate buffered saline (PBS) containing 5% (w/v) skimmed milk and 0.05% Tween-20 and then incubated with serotype X-specific monovalent grouping sera 7;8 (Denka Seiken, Japan), serotype 1a-specific monovalent typing sera I (Denka Seiken, Japan), or monovalent grouping sera 3;4 (Denka Seiken, Japan). The membrane was then washed with 1 × PBS containing 0.05% Tween-20, and incubated with a secondary anti-rabbit antibody labeled with the fluorescent IRDye™ 800 (Rockland). Fluorescence was detected using an Odyssey Infrared Imaging System (LI-COR).

### Identification of the integration site and orientation of SfX and SfI

Based on previous studies showing that the integration site of serotyping-conversion bacteriophages is conserved [15], a series of primers were designed that were located in genes *proA*, *yaiC*, *gtrI*, *gtrX*, *intI *and *intX *across the presumptive integration region to determine the site and order of integration using PCR: *proA*-F, ACAAAGCGAAATCATCCTCAA; *intI*-R, AGTGTTACAGGAAATGGGAGGC. *gtrI*-F, ATTGAACGCCTCCTTGCTATGC; *intX*-R, TACGGTGGCTGCGTGAGAA. *gtrX*-F, TACCTTGACCCGTTTCCG; and *yaiC*-R, GCAGGAAACCACCATCAACACC. PCR products were sequenced commercially to identify the integration site precisely.

## Authors' contributions

JX and QS designed the study, and co-drafted the manuscript. RL participated in the design of the study and preparation of the manuscript. YW participated in the construction of the new serotype. JW carried out the PCR amplification and DNA sequencing. XL performed the LPS Western-blot assay. SZ carried out the serological identification. PL participated in the phage induction and infection. CY and HJ participated in the isolation of clinical strains. YW participated in the sequence alignment. All authors read and approved the final manuscript.

## Supplementary Material

Additional file 1 **Supplementary figure**. DNA sequences of integration sites in 036, 036_X and 036_1d, and bacteriophages SfI and SfX. Sequences obtained by PCR and sequencing of junction regions using a series of primers across the integration site as described in the text. (A) *attB *in strain 036. (B) *attP *in phage SfI. (C) *attP *in phage SfX. (D) *attL *in strain 036_X. (E) *attR *in 036_X and 036_1d. (F) Sequence between phage SfI and SfX in strain 036_1d. Sequences in box are conserved DNA regions between genes; Underlined sequences are *tRNA-thrW*; Sequences in blue are *att *core sequence; Conserved genes flanking a given integration site are shaded and their transcription orientation is marked by an arrow. Click here for file
