# Supplementary material for: Genesis of a novel Shigella flexneri serotype by sequential infection of serotype-converting bacteriophages SfX and SfI
Source: BMC Microbiol. 2011 Dec 30;11:269. doi: 10.1186/1471-2180-11-269 (PMC3306764; doi:10.1186/1471-2180-11-269)
Supplement: Additional file 1 — Supplementary figure. DNA sequences of integration sites in 036, 036_X and 036_1d, and bacteriophages SfI and SfX. Sequences obtained by PCR and sequencing of junction regions using a series of primers across the integration site as described in the text. (A) attB in strain 036. (B) attP in phage SfI. (C) attP in phage SfX. (D) attL in strain 036_X. (E) attR in 036_X and 036_1d. (F) Sequence between phage SfI and SfX in strain 036_1d. Sequences in box are conserved DNA regions between genes; Underlined sequences are tRNA-thrW; Sequences in blue are att core sequence; Conserved genes flanking a given integration site are shaded and their transcription orientation is marked by an arrow. [file 1471-2180-11-269-S1.PDF]

>> *proA* >>

ACCATTCGTGCGTAAATAAAACCGGGTGATGCAAAAGCAGCCATTTGATTCACAAGGCCATTGACGCA
TCGCCCGGTTAGTTTTAACCTTGTCCACCGTGATTCACGTTCGTGAACATGTCCTTTCAGGGCCGATAT
AGCTCAGTTGGTAGAGCAGCGCATTCGTAATGCGAAGGTCGTAGGTTCGACTCCTATTATCGGCACCA
TTTAAATCAATAAGTTACACATCATTAGTACCTTC

<<putative phage *int* <<

A

<< *gtrIA* <<

TACAAATAACTTTAACATTTATTTAATCAATGAGCTCTGAAAGGCATGAAGTCTATCATCCAAGTCTCA
ATTGATCGATACTTGCTATGTCTGATGAGACAAAACTGAGACACATAAGGCCTCACAATGGCTTGCAA
GGCTTTACATGTTTTGATGTGGTGGGACGTGTGAGCGCAGTGTTGATGGGATAATCCTTTGAATTACA
AGCGGATTCTTATAATTCGTAATGCGAAGGTCGTAGGTTCGACTCCTATTATCGGCACCATTAAAATCA
ATAAGTTACACATCATTAGTACCTTC

<< *intI* <<

B

<< *gtrXA* <<

TGCAAATAGCTTTAACATTTCTTCTATCAGCTAATAATCAAAGGCATGAAGTCTATCATCCAAGTCTCAA
TCGATCGATACTTGCGGTAGTTGATGAGAAAAACTCTGGCACACAAATCTTTGCACTGGATTGCAAGG
CTTTGTGCTCTTCTGTGAATGTGTGGCTATATGTTTGAAGATCGTTGTGCCGTATTTGTGACATATATAT
GGCAACATCATTCATCAACTTTCTGTTTGTGCCATCAACTATATCTTAGTGAATGCGGTTAATGCTTGCT
AAAACAGATAGTTATGATTGGTGCTACAGATTCGTAATGCGAAGGTCGTAGGTTCGACTCCTATTATCG
GCACCATTTAAATCAATAAGTTACCTCGCATTTAAGTA

<< *intX* <<

C

>> *proA* >>

ACCATTCGTGCGTAAATAAAACCGGGTGATGCAAAAGCAGCCATTTGATTCACAAGGCCATTGACGCA
TCGCCCGGTTAGTTTTAACCTTGTCCACCGTGATTCACGTTCGTGAACATGTCCTTTCAGGGCCGATAT
AGCTCAGTTGGTAGAGCAGCGCATTCGTAATGCGAAGGTCGTAGGTTCGACTCCTATTATCGGCACCA
TTTAAATCAATAAG TTACCTCGCATTTAA

<< *intX* <<

D

<< *gtrXA* <<

AAATAGCTTTAACATTTCTTCTATCAGCTAATAATCAAAGGCATGAAGTCTATCATCCAAGTCTCAATCG
ATCGATACTTGCGGTAGTTGATGAGAAAAACTCTGGCACACAAATCTTTGCACTGGATTGCAAGGCTT
TGTGCTCTTCTGTGAATGTGTGGCTATATGTTTGAAGATCGTTGTGCCGTATTTGTGACATATATATGGC
AACATCATTCATCAACTTTCTGTTTGTGCCATCAACTATATCTTAGTGAATGCGGTTAATGCTTGCTAAA
ACAGATAGTTATGATTGGTGCTACAGATTCGTAATGCGAAGGTCGTAGGTTCGACTCCTATTATCGGCA
CCATTTAAATCAATAAGTTACACATCATTAGTACC

<<putative phage *int* <<

E

<< *gtrIA* <<

TAACTTTAACATTTATTTAATCAATGAGCTCTGAAAGGCATGAAGTCTATCATCCAAGTCTCAATTGATC
GATACTTGCTATGTCTGATGAGACAAAACTGAGACACATAAGGCCTCACAATGGCTTGCAAGGCTTTA
CATGTTTTGATGTGGTGGGACGTGTGAGCGCAGTGTTGATGGGATAATCCTTTGAATTACAAGCGGAT
TCTTATAATTCGTAATGCGAAGGTCGTAGGTTCGACTCCTATTATCGGCACCATTTAAATCAATAAGTTA
CCTCGCATTTAA

<< *intX* <<

F
